# Genomic Features and Niche-Adaptation of *Enterococcus faecium* Strains from Korean Soybean-Fermented Foods

**DOI:** 10.1371/journal.pone.0153279

**Published:** 2016-04-12

**Authors:** Eun Bae Kim, Gwi-Deuk Jin, Jun-Yeong Lee, Yun-Jaie Choi

**Affiliations:** 1 Department of Animal Life Science, College of Animal Life Sciences, Kangwon National University, Chuncheon, Kangwon-do, Republic of Korea; 2 Division of Applied Animal Science, College of Animal Life Sciences, Kangwon National University, Chuncheon, Kangwon-do, Republic of Korea; 3 Department of Agricultural Biotechnology, Seoul National University, Seoul, Republic of Korea; 4 Research Institute for Agriculture and Life Science, Seoul National University, Seoul, Republic of Korea; University Medical Center Utrecht, NETHERLANDS

## Abstract

Certain strains of *Enterococcus faecium* contribute beneficially to human health and food fermentation. However, other *E*. *faecium* strains are opportunistic pathogens due to the acquisition of virulence factors and antibiotic resistance determinants. To characterize *E*. *faecium* from soybean fermentation, we sequenced the genomes of 10 *E*. *faecium* strains from Korean soybean-fermented foods and analyzed their genomes by comparing them with 51 clinical and 52 non-clinical strains of different origins. Hierarchical clustering based on 13,820 orthologous genes from all *E*. *faecium* genomes showed that the 10 strains are distinguished from most of the clinical strains. Like non-clinical strains, their genomes are significantly smaller than clinical strains due to fewer accessory genes associated with antibiotic resistance, virulence, and mobile genetic elements. Moreover, we identified niche-associated gene gain and loss from the soybean strains. Thus, we conclude that soybean *E*. *faecium* strains might have evolved to have distinctive genomic features that may contribute to its ability to thrive during soybean fermentation.

## Introduction

*Enterococcus faecium* is a Gram-positive bacterium found in the gastro-intestinal tracts (GIT) of animals [1], fermented foods [2,3], and a variety of other environments [4]. *E*. *faecium* is a lactic acid bacterium, which produces lactic acid as a final product of carbohydrate fermentation [5], and it may have hypothetical roles in the early stages of soybean fermentation [3,6]. Some strains of *E*. *faecium* have beneficial roles in GIT health as commensal or probiotic bacteria [7]; others are associated with nosocomial infections such as bacteremia and endocarditis in humans [1]. Recently, certain *E*. *faecium* strains have emerged as critical hospital pathogens due to their resistance to vancomycin treatments [1].

Although the pathogenicity of *E*. *faecium* has not been yet fully defined, many antibiotic resistance (AR) genes and virulence factors (VF) have been identified [1]. Mobile genetic elements (ME) contribute to horizontal transfer of co-localized AR and VF genes [8]. AR, VF, and ME genes are more frequently found in clinically isolated *E*. *faecium* strains than community-associated non-clinical strains [8]. Moreover, comparative genomic analysis of *E*. *faecium* strains revealed different lineages that represent clinical and non-clinical strains [8–10].

Many Korean foods have long been prepared based on fermentation of soybeans. Fermented soybean blocks (Meju), fermented soybean paste (Doenjang), and soy sauce (Ganjang) are representative fermented soybean foods in Korea. A recent study showed that *Enterococcus* spp were found at considerable levels in fermented soybeans at the early stage of fermentation [6], and *E*. *faecium* strains were frequently isolated from the fermented products [3]. Despite the important roles of *E*. *faecium* in soybean fermentation, genomic features and contents of *E*. *faecium* have never been evaluated by using genomic information. Here, we sequenced genomes of 10 *E*. *faecium* strains isolated from fermented soybeans to characterize their genomic features.

## Materials and Methods

### *E*. *faecium* strains

The 10 *E*. *faecium* strains used in this study were obtained from a Microorganism Collection, Korean Agricultural Culture Collection (KACC). They were all isolated from fermented soybean products as listed in Table 1. To avoid clonality or geographical relatedness among the 10 strains, we selected them from the products of seven independent commercial companies.

**Table 1:** Ten soybean *E*. *faecium* strains and genomes sequenced in this study.

Strains^a^	Origins^b^ / Producers	Genome (bp)	Contigs	Max Contig (bp)	N50 (bp)	GC (%)	Accession^c^
15689	Doenjang / A	2,824,234	128	213,108	72,492	38.0	LBIT00000000
15960	Doenjang / B	2,692,315	93	185,163	71,626	38.1	LDNE00000000
15962	Doenjang / C	2,719,801	99	216,188	65,696	38.2	LDNF00000000
15700	Ganjang / A	2,852,054	123	213,345	81,177	38.0	LDNC00000000
15711	Meju / A	2,781,534	145	175,610	61,421	38.0	LDND00000000
16076	Meju / D	2,719,289	73	354,186	104,708	38.1	LDNG00000000
16093	Meju / E	2,585,336	96	197,388	57,918	38.1	LDNH00000000
16097	Meju / F	2,630,779	64	194,680	104,580	38.2	LDNI00000000
16100	Meju / G	2,837,155	104	159,513	83,784	38.0	LDNJ00000000
16106	Meju / B	2,673,905	74	268,871	107,917	38.0	LDNK00000000

^**a**^Strain numbers assigned by KACC

^**b**^Doenjang, fermented soybean paste; Ganjang, soy source; Meju, fermented soybean block

^**c**^NCBI GenBank Accession Numbers. Both chromosome and plasmids are included.

### Preparation of Genomic DNA Library and Sequencing

Bacterial cells were harvested from overnight BHI (Brain-Heart Infusion) broth culture. Harvested cells were washed two times with 1X PBS buffer. The cells were further processed to extract genomic DNA using G-spin Genomic DNA Extraction Kit (iNtRON Biotechnology, Cat #17121, South Korea). Genomic DNA was fragmented using NEBNext dsDNA Fragmentase (NEB, Cat #M0348S, MA, USA). The fragmented DNA was further processed to construct a genomic DNA library using NEBNext Ultra DNA Library Prep Kit for Illumina (NEB, Cat # E7370S, MA, USA). Genomic DNA libraries were constructed with ~350-bp inserts and sequenced by Illumina HiSeq2500 for 100 bp paired-end reads.

### Genome Assembly and Annotation

Sequenced reads were quality-filtered using in-house Perl scripts [11]. In brief, when 95% of nucleotide bases in a read were given a quality score over 31 (Illumina 1.8+) and the read length was ≥70 bp, the read was used for genome assembly. The filtered paired and single reads were assembled using Ray 2.3.1 [12] with a k-mer size of 31 bp. The assembled draft genome sequences were uploaded to an annotation server, RAST [13] with default options for bacteria.

### Genome Comparison and Strain Clustering

For the ortholog collection, *E*. *faecium* nucleotide sequences were downloaded on 07/03/2014 from the NCBI GenBank database. We inspected protein coding sequences (CDS) from GenBank information using in-house Perl scripts. In brief, we excluded premature stop codons, codon shifts by deletions and insertions, errors in CDS length, etc. as shown previously [8]. CDS were extracted from NCBI *E*. *faecium* nucleotides and our 10 annotated *E*. *faecium* genomes. The CDS were clustered based on the sequence similarity as suggested previously [8]. In brief, CDS were mutually aligned to highly similar CDS to generate CDS clusters using high-throughput sequence alignment software, GASSST [14] under the parameters of ≥90% sequence identity and sensitivity level 5 (maximum). Each collection of clustered CDS was further assembled to make a consensus orthologous CDS. From the clusters, a total of 13,820 orthologous CDS were finally defined. Each CDS was examined to determine gene presence/absence. For strain clustering, we used genomes of 51 clinical and 52 non-clinical *E*. *faecium* strains (S1 Table) that were available from NCBI. Strain clustering analysis was conducted based on the presence/absence of each CDS as previously suggested [8]. Briefly, distance between strains was calculated according the Euclidean distance method. Existence of clades was statistically confirmed by 1,000 times re-sampling using the Pvclust R package [15].

### Phylogenetic Analysis

To analyze clonal relatedness among soybean strains and assign their lineage among 113 *E*. *faecium* strains, we collected single nucleotide polymorphisms (SNPs) from the 113 strains. We used 990 core genes that are commonly present in all genomes. Due to incomplete genome assemblies, 100 core genes are excluded from SNP selection. To collect core gene sequences from each genome, ortholog sequences were aligned to each genome using GASSST under the 75–100% sequence similarity option. The aligned regions in each genome were extracted and collected for SNP selection. The collected core gene sequences from each genome were aligned among 113 strains using the alignment tool MUSCLE [16]. On the basis of the core gene alignment, we could detect SNPs. All of the SNPs in the core genes were further used to construct phylogenetic trees using the bioinformatics tool MEGA [17]. The evolutionary history and distance were inferred and computed using the Neighbor-Joining method [18] with bootstrap tests [19] and the Maximum Composite Likelihood method [20], respectively.

### Detection of VF, AR, ME, and BA Genes

As previously defined [8], 34 VF genes, 748 AR orthologs, and ME genes were collected based on literature review [8], ARDB database [21], and sequence annotations [8], respectively. Bacteriocin-associated (BA) genes including both bacteriocins and their regulatory genes were collected based on known bacteriocin genes found in a bacteriocin database (BAGEL, http://bagel.molgenrug.nl/) [22]. Each genome was screened for the presence of these genes as described above.

### Detection of Soybean Niche-Specific Genes

The frequency of each orthologous CDS was counted for *E*. *faecium* from all three different origins; soybean origin, non-soybean non-clinical origin, and clinical origin. Genes with higher frequency in the soybean origin than other origins were regarded as soybean-specific. Significance of different frequencies was evaluated by Fisher's Exact Test at *P* < 0.05.

## Results

### Relationships of Soybean *E*. *faecium* Strains

**Fig 1:**
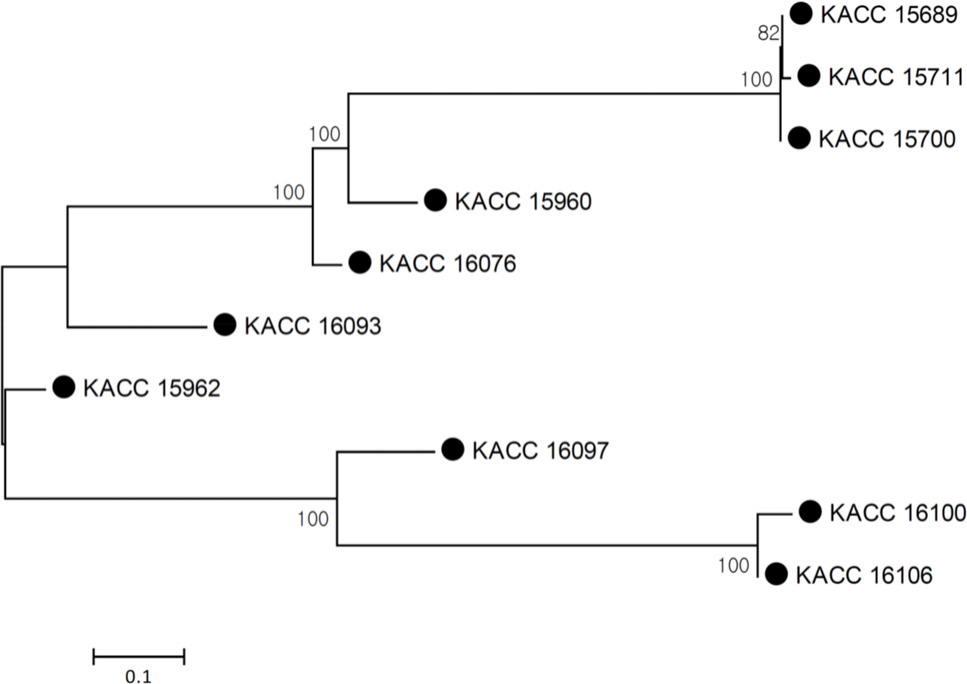
Evolutionary relationships of 10 soybean strains based on 8,850 SNPs from 990 core genes. The optimal tree with the sum of branch length = 2.15 is shown. The percentage of replicate trees in which the associated taxa clustered together in the bootstrap test (1000 replicates) are shown next to the branches. The tree is drawn to scale, with branch lengths in the same units as those of the evolutionary distances used to infer the phylogenetic tree. The evolutionary distances are in the units of the number of base substitutions per site.

Before we describe the differences between 10 soybean strains and others from different origins, we need to demonstrate whether any specific strains are clonally related, which may affect subsequent comparisons. In total, 8,850 SNPs from 990 core genes, which are commonly found in 113 strains, were identified among the 10 soybean strains. We constructed a phylogenetic tree (Fig 1) that was based on these SNPs. While seven soybean strains are not closely related, three other strains (KACC 15689, KACC 15700 and KACC 15711) were very close. The three closely related strains were isolated from different products produced by the same company. However, we also identified 125 SNPs among these three strains. This observation indicates that 10 soybean strains are not highly biased and are appropriate for comparison between 10 soybean strains and others from different origins.

### Genome Sequencing and Genome Assembly

**Fig 2:**
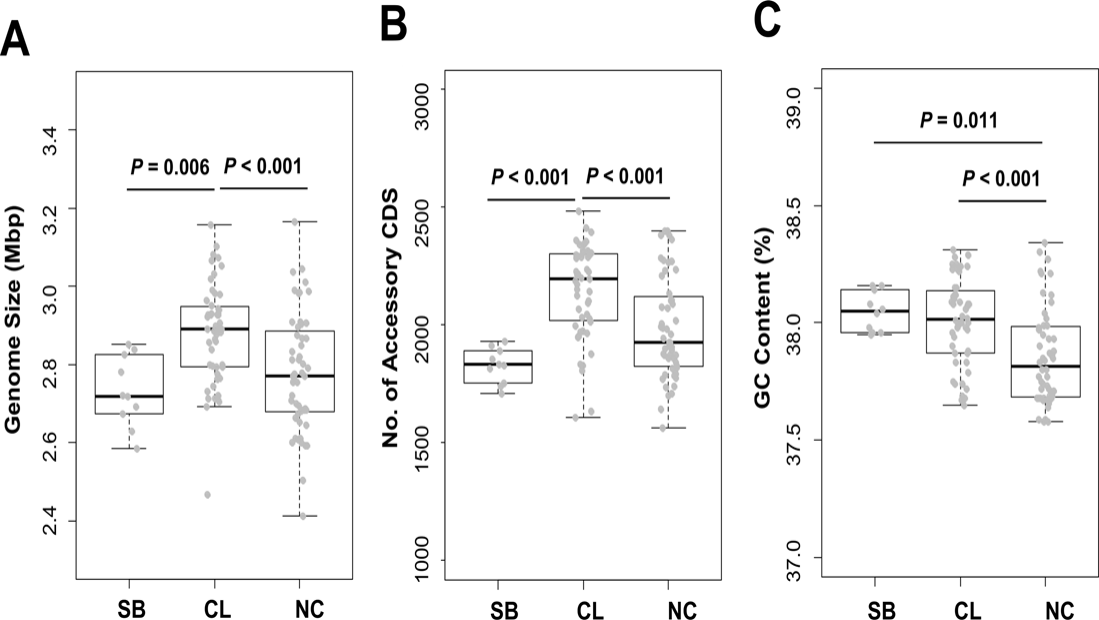
Genome size, number of accessory CDS, and GC content (%) of soybean strains. Genome sizes (A), accessory genes (B), and GC content (C) were compared among soybean (SB), clinical (CL) and non-clinical (NC) groups. Each gray spot indicate a single *E*. *faecium* strain.

Ten *E*. *faecium* strains from fermented soybean were sequenced and assembled to obtain draft genomes (Table 1). The numbers of contigs ranged from 64 to 145. The average genome size of the strains was 2.73±0.09 Mbp, and the GC content ranged from 37.95% to 38.16%. The average genome size of soybean-isolated strains (n = 10, Fig 2A) was significantly smaller than that of the clinical strains (n = 51, 2.88±0.13 Mbp, *P* = 0.006), but not compared to that of non-clinical strains (n = 52, 2.78±0.15 Mbp). From our pan-genome analysis, we identified 1,090 core CDS that are found in all 113 *E*. *faecium* genomes (10 soybean strains, 51 clinical strains, and 52 non-clinical strains). The number of accessory CDS that were found only in some strains was compared among the three groups (Fig 2B). Soybean-isolated strains have a total of 1,828±74 accessory CDS, which are significantly fewer than in clinical strains (2,149±197, *P*<0.001). Non-clinical strains (1,985±217) also have the higher numbers of accessory CDS compared to clinical strains (*P*<0.001). The average GC content of soybean-isolated strains (38.05±0.08%, Fig 2C) was similar to that of clinical strains (38.01±0.19%), and GC content was higher in both groups compared to non-clinical strains (37.86±0.20%, *P* = 0.011 and *P*<0.001).

### Lineage of *E*. *faecium* Strains from Soybean-Fermented Foods

**Fig 3:**
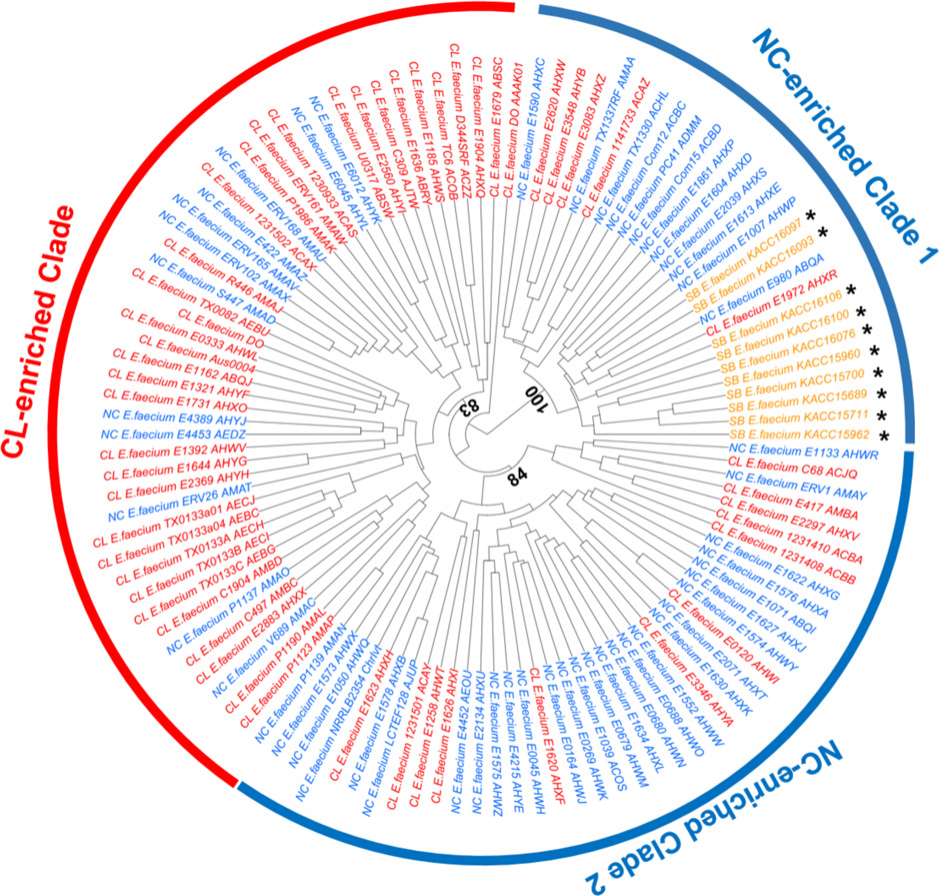
Hierarchical clustering of the 113 *E*. *faecium* strains used in this study. Based on ortholog presence/absence, clusters were obtained. Asterisks indicate the 10 soybean strains (Prefix: SB). Clinical and non-clinical strains were indicated by different prefixes, CL and NC, respectively. Approximately unbiased probabilities for the bootstrapping are shown in the center of the dendrogram.

**Fig 4:**
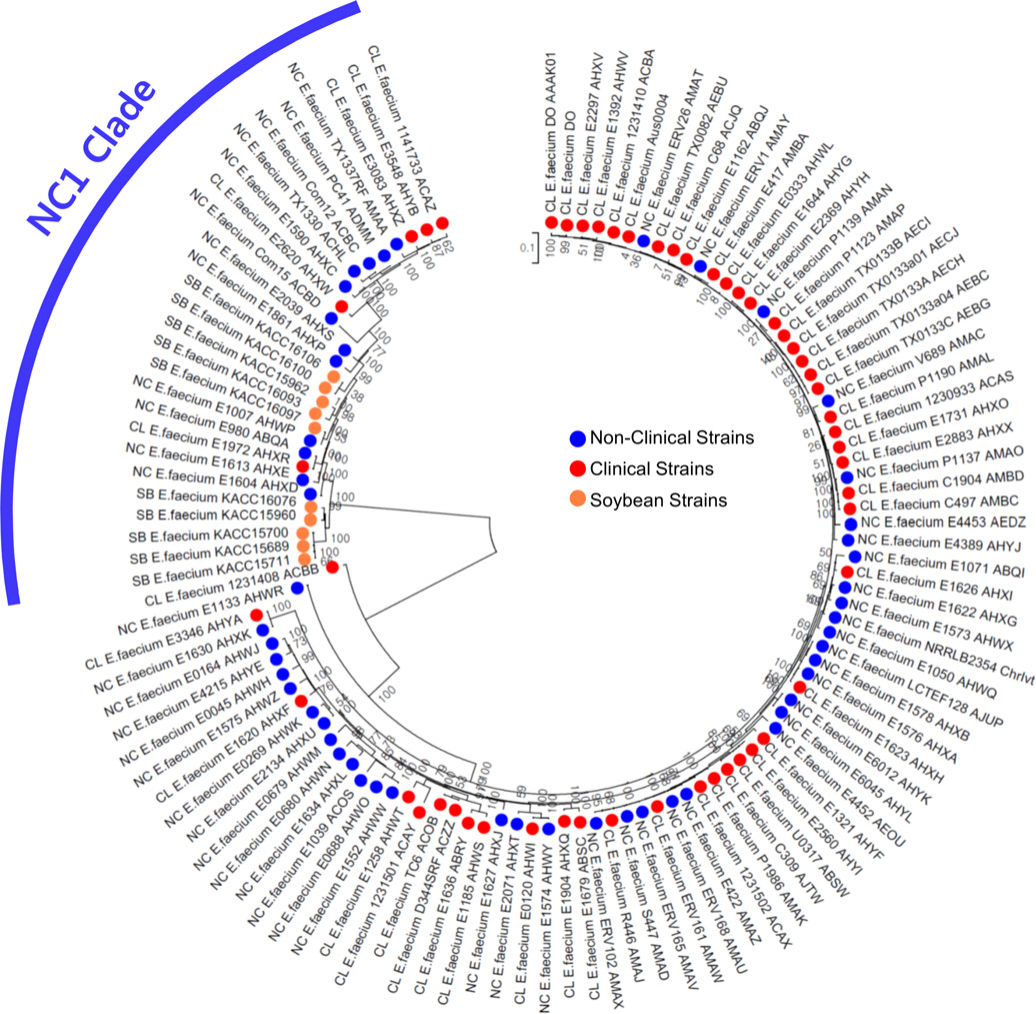
Evolutionary relationships of 113 strains based on 59,739 SNPs from 990 core genes. The optimal tree with the sum of branch length = 3.21 is shown. The tree is drawn to scale, with branch lengths in the same units as those of the evolutionary distances used to infer the phylogenetic tree. The evolutionary distances are in the units of the number of base substitutions per site.

According to previous studies [8,11], non-clinical strains belong to two major groups called the NC1 and NC2 clades; these are distinct from the CL clade, which is enriched with clinical strains. Here, we examined where the tested soybean strains were positioned by a hierarchical clustering analysis. Based on the presence or absence of orthologous CDS found in our pan-genome analysis, the 113 *E*. *faecium* genomes were positioned as shown in Fig 3. As expected, the soybean-isolated strains were positioned in NC-enriched clade 1 but not in NC-enriched clade 2, which clustered together with a major CL-enriched clade. The NC-enriched clades and CL-enriched clade were matched to previously defined clades, NC1, NC2 and CL [8,11]. We also identified that 10 soybean strains are positioned at the NC1 clade on a phylogenetic tree based on 59,739 SNPs detected from 990 core genes (Fig 4). This result indicates that soybean strains are genetically much closer to non-clinical strains than to clinical strains.

### Prevalence of AR, VF, ME, and BA genes

**Fig 5:**
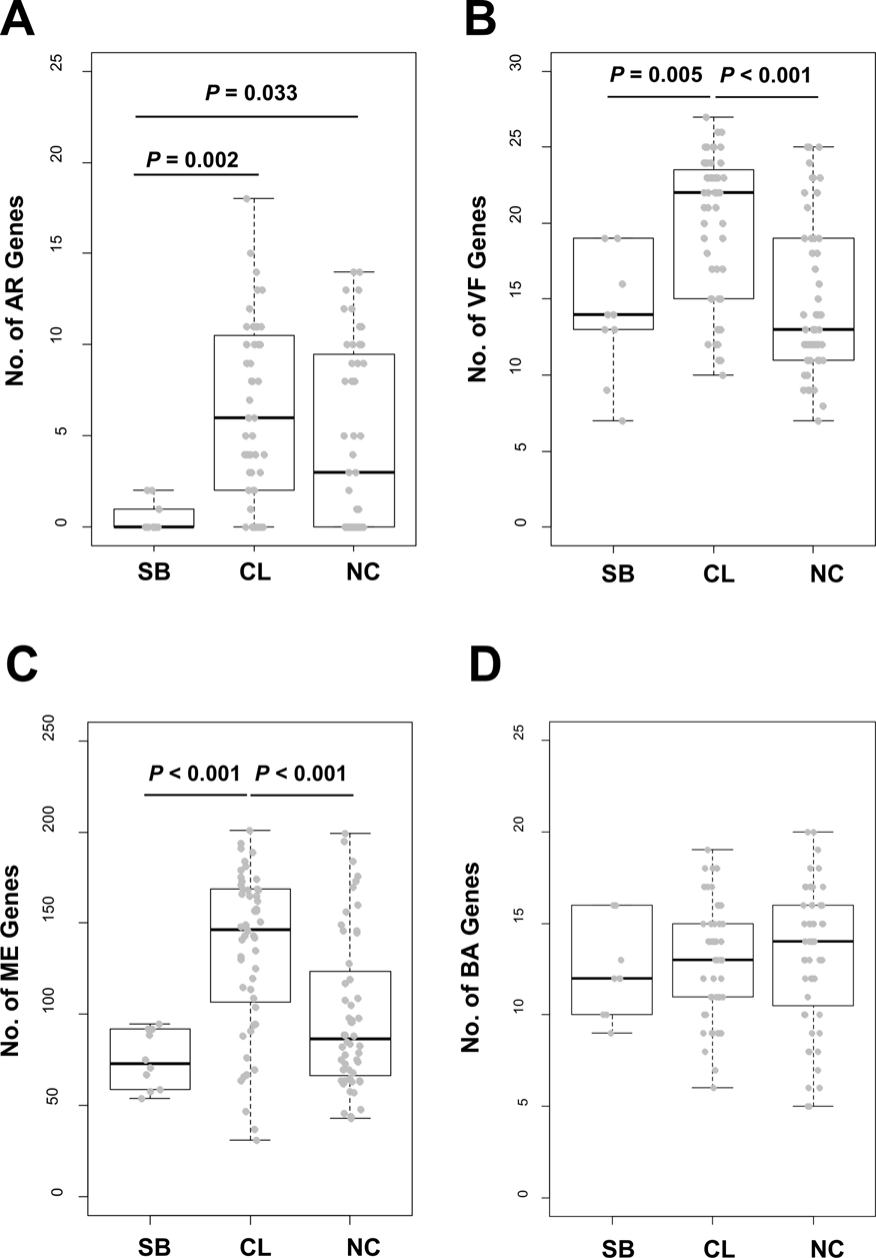
Frequencies of AR, VF, ME, and BA genes. Frequencies of four types of genes, AR (A), VF(B), ME (C) and BA (D), were compared among soybean (SB), clinical (CL) and non-clinical (NC) groups. Statistical significance was examined using Tukey's HSD (Honestly Significant Difference). Non-significant comparisons were omitted (*P*>0.05). Each gray spot indicate a single *E*. *faecium* strain.

AR, VF, ME, and BA genes contribute not only to the pathogenicity of *E*. *faecium* species [1] but also to environmental adaptation [8,11,23,24]. To better understand soybean *E*. *faecium* strains, we analyzed the prevalence of these genes (Fig 5). The soybean strains have fewer AR genes than both clinical and non-clinical strains. Only two AR genes, *bacA* and *tetU*, were found in certain soybean strains although a role of *tetU* as a resistance gene is under debate [25], and no vancomycin resistance genes were found. The soybean strains also have fewer VF and ME genes than the clinical strains but have a similar number of VF and ME genes as non-clinical strains [8,11]. Some VF genes (*efmA*, *acm*, *sgrA*, *cad*, and *cbh3*) were found in all soybean strains; however, the 3 major VF genes (*IS16*, *hyl*_*efm*_, and *esp*) that are used for safety assessment in Europe [26] were not found in any soybean strain. The number of BA genes was not significantly different among the three groups. These results suggest that the genomic content of soybean strains of *E*. *faecium* is more similar to non-clinical strains than to the clinical strains.

### Niche-Enriched Genes in Soybean *E*. *faecium* Strains

**Fig 6:**
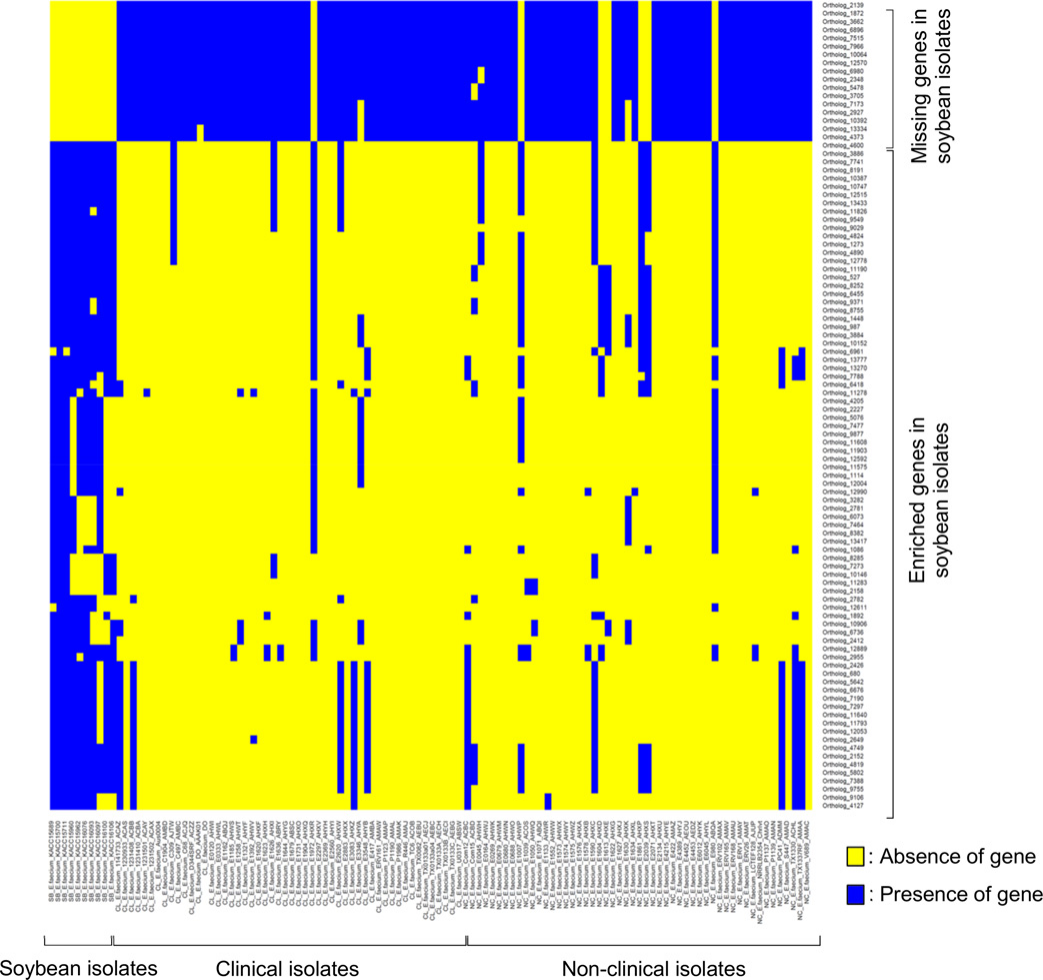
Niche-specific gene gain and loss in soybean *E*. *faecium* strains. Enriched or missing genes in soybean strains were identified and sorted according to *P* values (S2 Table). If *P* values of a gene both between soybean and clinical strains and between soybean and non-clinical strains were less than 0.005 (Fisher’s exact test), the gene was regarded as enriched or missing in the soybean strains.

Although the genomic patterns of soybean *E*. *faecium* strains are similar to non-clinical strains as described above, we expected that they would have their own niche-enriched genes that are not frequently found in isolates of other origins. We identified not only such niche-enriched genes, but also missing genes in soybean *E*. *faecium* strains (Fig 6 and S2 Table). In total, 81 niche-specific genes were enriched in soybean strains and they were significantly fewer in the clinical (*P*<0.005) and non-clinical strains (*P*<0.005). Among the 81 genes, 6 gene clusters were identified (S1 Fig and S2 Table). Three clusters (Clusters 1, 3, and 5) were completely aligned to three known *E*. *faecium* plasmids (GenBank Accession No. CP013995, CP006030, and DQ198088), and another cluster (Cluster 4) was found not only in many *E*. *faecium* contigs but also in *Enterococcus faecalis* (GenBank Accession No. KJ756557) and *Enterococcus durans* (GenBank Accession No. CP012367). Clusters 1, 4, 5, and 6 are related to sugar metabolism, cell wall-associated function, the enterocin AS-48 system, and mannose/fructose transportation, respectively. We identified 17 genes that were frequently found in *E*. *faecium* from both clinical and other origins but were frequently missing from the soybean *E*. *faecium* strains. Among the 17 genes, we identified two major clusters (S1 Fig and S2 Table). Clusters 7 and 8 are related to arabino-furanose metabolism and mannose/fructose transportation, respectively. These results suggest that soybean *E*. *faecium* strains have niche-specific and nutrition/environment-adaptive genomic features, which distinguish them from *E*. *faecium* from other origins.

## Discussion

Although *E*. *faecium* strains are frequently found in soybeans [27,28] and soybean-fermented foods [3,6], to our knowledge, the genomes of soybean-originated *E*. *faecium* have never been investigated. Here, we sequenced the genomes of *E*. *faecium* strains from fermented soybean foods and compared them to the genomes of *E*. *faecium* isolated from other sources.

Several genomic studies on *E*. *faecium* strains showed differences in *E*. *faecium* lineages–clinical and non-clinical clades [8,11,29,30]. *E*. *faecium* has been recognized as commensal and/or probiotic species. However, pathogenic phenotypes have emerged in certain clinical *E*. *faecium* strains due to overuse of antibiotics in hospitals [1], and these pathogenic strains show different genomic and phenotypic features because they have obtained niche-adapted genetic resources such as AR and VF genes to survive in the presence of antibiotics and to grow in hospitalized patients [8,11]. In this study, we identified that soybean *E*. *faecium* strains are closely clustered together with non-clinical strains (Fig 3). Unlike clinical strains, the soybean strains may not require extra AR and VF genes to survive in the soybean environments because the fermented soybean environments may be conducive for growth and are rich in nutrients that are produced due to the activity of *Bacillus* sp. [31,32].

Genomic GC contents of the soybean strains were slightly higher than those of CL and NC strains. The higher GC contents of the soybean strains were influenced by accessory genes, which are lower in GC content than core genes (Core: 39.14%, Accessory: 38.13%, *P* < 0.0001). The soybean strains (1927.5±74.4 genes) have a lower number of accessory genes than CL and NC strains (2178.5±292.0 genes), which differentially influenced the GC contents of the entire genome. That is, lower-GC accessory genes are more abundant in NC and CL strains than in soybean strains. More specifically, ME genes (37.05%) were lower in GC content than other genes (38.25%, *P* < 0.001), and there were significantly fewer such ME genes in the soybean strains (75.2±15.8 genes) than in both CL and NC strains (117.4±46.5 genes, *P* < 0.001). However, soybean-enriched genes may have a limited effect on the higher GC contents of the soybean strains, although they are significantly lower in GC content (34.20%) than other genes (38.23%, *P* < 0.001). For now, the reason for the slightly higher GC contents in soybean strains is not fully understood. Other types of accessory genes or codon usage bias may play a role in such a high GC content, which must be clarified in future studies.

Recently, the safety of *E*. *faecium* for use in food products has been questioned, due to widespread antibiotic resistance in this species [26]. *E*. *faecium* used in soybean fermentation lacks virulence and antibiotic resistance genes and therefore safe for human use, as was previously shown for the dairy strain NRRL B-2354 [11].

We didn’t observe any significant differences in the number of BA genes between soybean-isolated *E*. *faecium* and other strains from different origins (Fig 5D). These data indicate either that soybean *E*. *faecium* may have antimicrobial activities similar to *E*. *faecium* from different origins or that clinical strains might have maintained their BA genes because of unknown ecological reasons such as competitive exclusion against other bacteria in the infected patients. BA genes associated with only immunity to enterocin AS-48 [33], which is antimicrobial against *Bacillus subtilis*, a major species in fermented soybean foods [34], were identified in soybean strains (S2 Table).

Soybeans are distinctive environments that are different from other plant-derived food sources and from the animal guts because they are richer in plant proteins. We hypothesized that soybean *E*. *faecium* strains might have been influenced by such nutrient-rich environments. We identified niche-enriched or missing genes in soybean strains compared with other *E*. *faecium* strains. Genes associated with fructose and mannose metabolism were simultaneously found in both enriched and missing genes (S2 Table). The missing functions mediated by Cluster 8 (mannose/fructose metabolism) may have been complemented by other similar carbohydrate metabolic genes (Cluster 6) in the soybean environments. Gain and loss of nutrient transporting genes, which are associated with PTS systems and permeases, may have also been influenced by the distinctive nutritional environments that are formed during protein and carbohydrate degradation mediated by *B*. *subtilis* [35,36]. As clusters 1 and 2 have integrases, they may act as mobile genetic elements. Similarly, Cluster 4 may be transferred among *E*. *faecium*, *E*. *faecalis*, and *E*. *durans*, which are found in soybean-fermented foods [3]. As many niche-enriched genes encode hypothetical proteins, further studies are required to clarify their functions and roles in soybean fermentation.

In this study, we characterized the genomes of 10 soybean *E*. *faecium* strains to identify genomic features and contents that distinguished them from *E*. *faecium* from other origins. Our results suggest that soybean-isolated *E*. *faecium* strains are closely related to non-clinical *E*. *faecium* strains, have distinct genomic features from the clinical strains, and have evolved to have niche-associated genomic contents. We hope that our findings will contribute to a better understanding of the role of *E*. *faecium* in soybean fermentation.

## Supporting Information

S1 Fig Clusters of niche-enriched or missing genes in soybean *E*. *faecium* strains. Cluster sequences were blasted to NCBI nr or WGS sequence database.
(DOC)

S1 Table Clinical and non-clinical E. faecium strains used in this study. (XLS)

S2 Table Frequency of genes in soybean (SB), clinical (CL), and non-clinical (NC) isoates. (XLS)
