# Supplementary material for: Genomic Features and Niche-Adaptation of Enterococcus faecium Strains from Korean Soybean-Fermented Foods
Source: PLoS One. 2016 Apr 12;11(4):e0153279. doi: 10.1371/journal.pone.0153279 (PMC4829236; doi:10.1371/journal.pone.0153279)
Supplement: S1 Fig — Cluster sequences were blasted to NCBI nr or WGS sequence database. (DOC) [file pone.0153279.s001.doc]

A. Cluster1


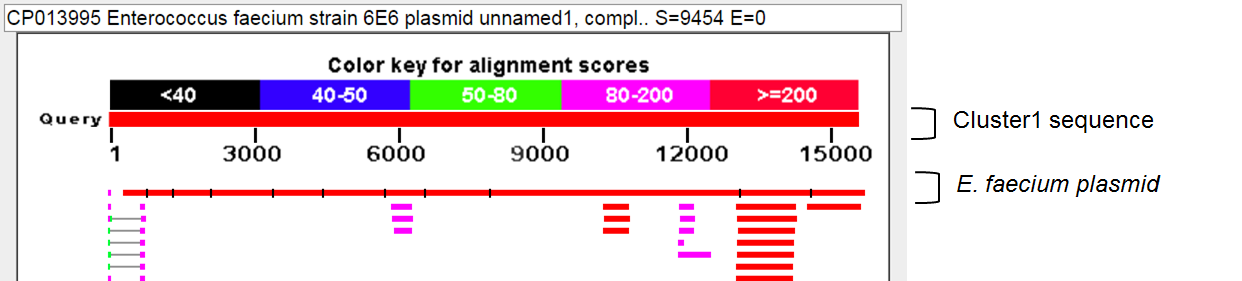


B. Cluster2


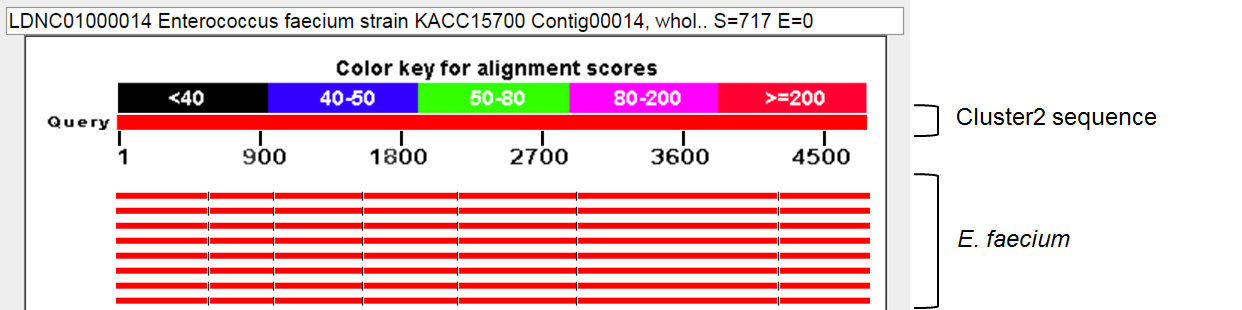


C. Cluster3


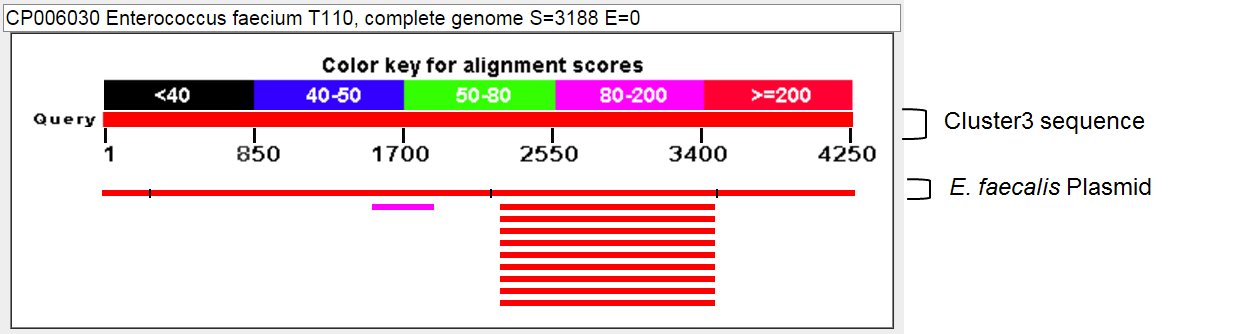


D. Cluster4


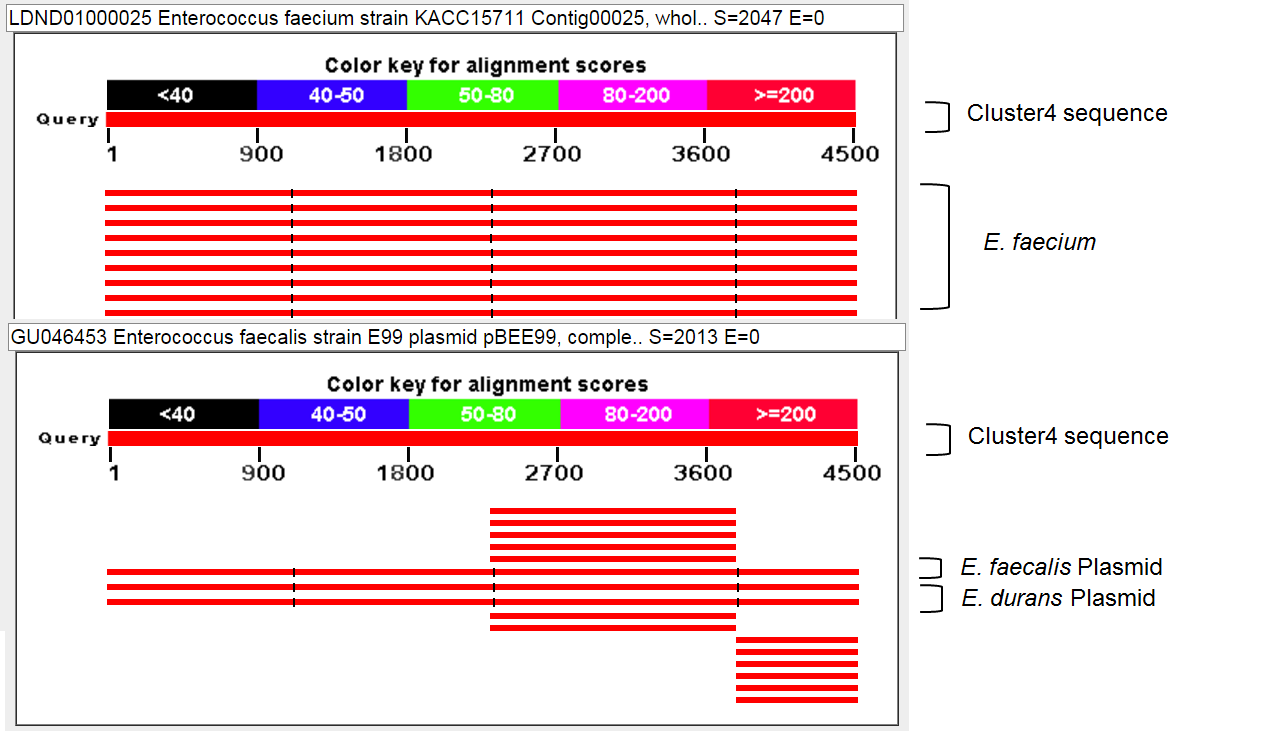


E. Cluster5


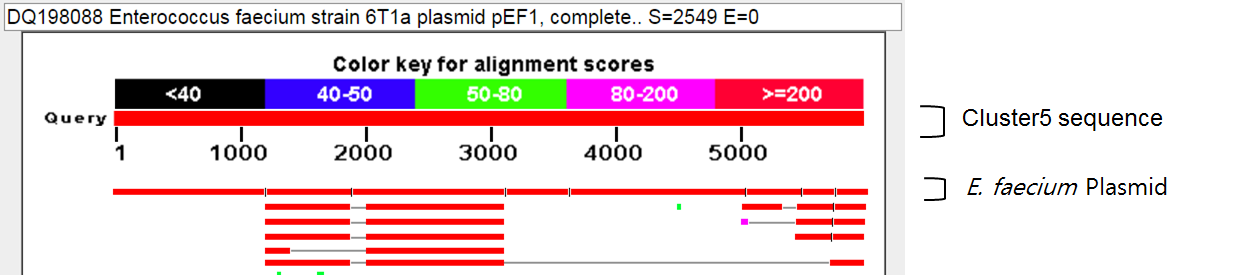


F. Cluster6


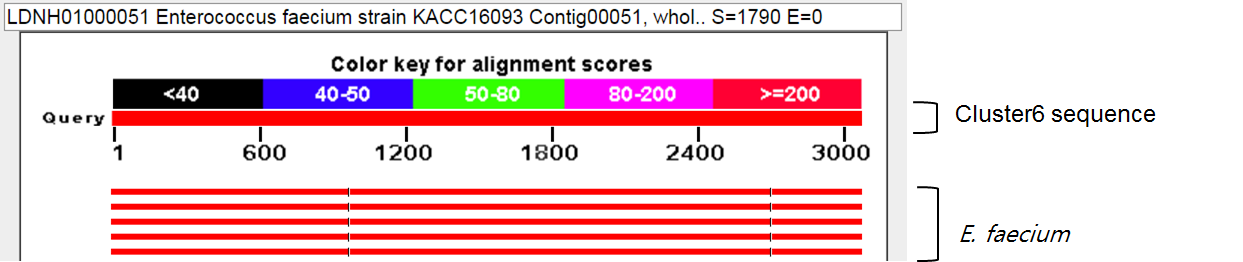


G. Cluster7


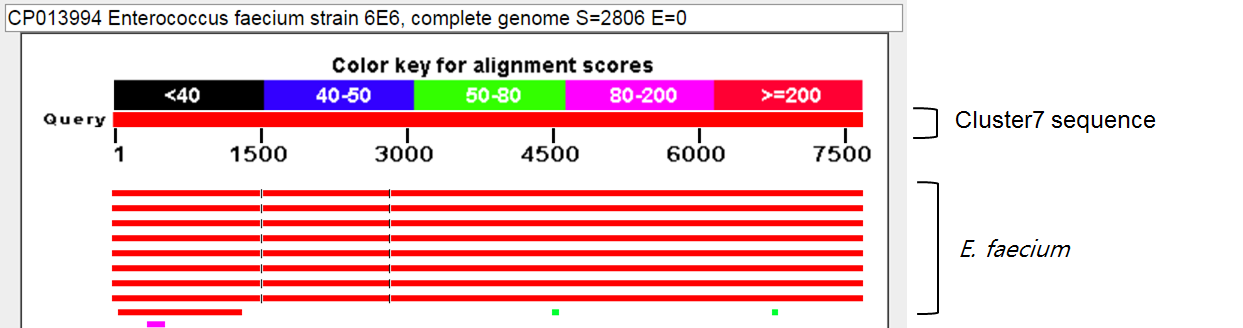


H. Cluster8


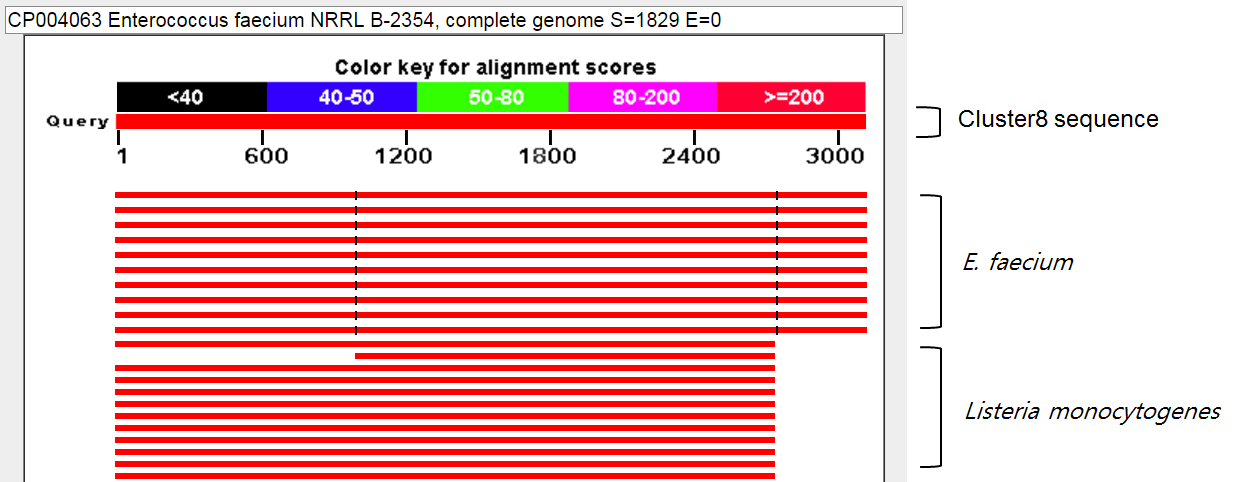


**S1 Fig**. **Clusters of niche-enriched or missing genes in soybean *E. faecium* strains.**

Cluster sequences were blasted to NCBI nr or WGS sequence database.
